# Add-on neurological benefits of antiviral therapy in HCV patients with chronic kidney disease — a nationwide cohort study

**DOI:** 10.1186/s12876-017-0653-2

**Published:** 2017-08-16

**Authors:** Ming-Shyan Lin, Tien-Hsing Chen, Wey-Yil Lin, Chi-Hung Liu, Yung-Yu Hsieh, Wen-Nan Chiu, Chih-Hsiang Chang, Mei-Yen Chen, Chang-Min Chung, Yu-Sheng Lin

**Affiliations:** 1 Department of Cardiology, Department of Internal Medicine, Chang Gung Memorial Hospital, Yunlin, Taiwan; 2 Department of Cardiology, Department of Internal Medicine, Chang Gung Memorial Hospital, Chang Gung University College of Medicine, Keelung, Taiwan; 3 Stroke Center and Department of Neurology, Chang Gung Memorial Hospital, Linkou Medical Center, Chang Gung University College of Medicine, Taoyuan, Taiwan; 4 Department of Hepato-Gastroenterology, Chang Gung Memorial Hospital, Chiayi, Taiwan; 5 Department of Hepato-Gastroenterology, Chang Gung Memorial Hospital, Yunlin, Taiwan; 6 Department of Nephrology, Kidney research center, Chang Gung Memorial Hospital, Chang Gung University, College of medicine, Taoyuan, Taiwan; 7 College of Nursing, Chang Gung University of Science and Technology (CGUST), Taoyuan, Taiwan; 8 Department of Nursing, Chang Gung University, Taoyuan, Taiwan; 9 School of Traditional Chinese Medicine, College of Medicine, Chang Gung University, Taoyuan County, Taiwan; 10 Department of Cardiology, Chiayi Chang Gung Memorial Hospital, 6, Sec. West Chai-Pu Road, Pu-TZ City, Chai Yi Hsien 61363 Taiwan

**Keywords:** Chronic kidney disease, Hepatitis C virus, Hemorrhagic stroke, Pegylated interferon

## Abstract

**Background:**

Hepatitis C virus (HCV)-infected patients with chronic kidney disease (CKD) have rarely been studied because they rarely accept interferon-based therapy (IBT) and have been difficult to follow up. We investigated long-term outcomes of IBT on the population.

**Methods:**

This population-based cohort study used the Taiwan National Health Insurance Research Database as its data source. HCV patients diagnosed with CKD between Jan. 1, 2003, and Dec. 31, 2013, were selected. They were then divided into two groups based on whether they had undergone IBT. All-cause mortality, acute myocardial infarction (AMI), ischemic stroke (IS), hemorrhagic stroke, and new-onset dialysis were evaluated using a Cox proportional hazard regression analysis after propensity score matching.

**Results:**

We enrolled 9872 HCV patients with CKD: 1684 patients in the treated cohort and 8188 patients in the untreated cohort. The annual incidence of all-cause mortality (19.00 vs. 42.89 events per 1000 person-years; *p* < 0.001) and the incidences of hemorrhagic stroke (1.21 vs. 4.19 events per 1000 person-years; *p* = 0.006) were lower in the treated cohort. New-onset dialysis was also lower in the treated cohort (aHR: 0.31; 95% CI: 0.20–0.48; *p* < 0.001).

**Conclusion:**

Antiviral therapy might provide protective benefits on all-cause mortality, hemorrhagic stroke, and new-onset dialysis in HCV-infected patients with CKD.

**Electronic supplementary material:**

The online version of this article (doi:10.1186/s12876-017-0653-2) contains supplementary material, which is available to authorized users.

## Background

Hepatitis C virus (HCV), which has a 2.8 ~ 3.0% prevalence worldwide, is strongly associated with major liver decompensation [1]. Chronic hepatitis C viral infection (CHC) is also strongly associated with end-stage renal disease (ESRD) [2] via extrahepatic metabovascular and immunological complications such as vasculitis, cryoglobulinemia, and glomerulonephritis [3]. The incidence of CHC is 10–50% in dialysis patients and kidney transplantation (KT) recipients [4, 5]. Although chronic kidney disease (CKD) poses a risk of cardiovascular disease and a higher mortality rate [6], HCV comorbid with CKD threatens even worse survival because of more comorbidities, more cardiovascular events, drug intolerance, and limited therapeutic responses.

Pegylated interferon-based therapy (IBT), a combination of pegylated interferon plus ribavirin, protected HCV patients against liver cirrhosis and hepatocellular carcinoma (HCC) [7], and successful viral eradication also reduced cardiovascular events and late dialysis in HCV patients without CKD [8, 9]. Additionally, IBT had satisfactory improvement of proteinuria and renal function in more than half HCV patients with cryoglobulinemic glomerulonephritis [10]. One meta-analysis also reported more than one half of dialysis patients with CHC had virological response to IBT [11]. Therefore, Kidney Disease Improving Global Outcomes (KDIGO) guidelines [12] recommended that antiviral therapy should be individual and mandated for most CKD patients with chronic HCV. Moreover, the efficacy of IBT in HCV patients with comorbid CKD and on hemodialysis remains challenging because of drug intolerance, anemia, and potential infection from adverse toxic effects [13]. Despite the big advances of new directacting antiviral (DAA) agents for HCV patients with advanced renal impairment [14], interferon-free therapy is still dependent upon specific genotypes or subtypes, the stage of CKD or dialysis, and the reactivation of a hepatitis B virus coinfection [15]. Moreover, DAAs might be unavailable in some parts of the world because of high price.

Our review of the literature revealed no studies on the effect of IBT on hepatic and extrahepatic complications in HCV patients with CKD and on dialysis. Thus, we investigated the benefits of IBT on all-cause mortality and extra-hepatic outcomes in HCV patients with comorbid CKD.

## Methods

### Data source

The data source for this population-based longitudinal cohort study is the Taiwan National Health Insurance Research Database (NHIRD), which has been prospectively recording claim data of all forms of healthcare service in Taiwan since 1995 [16]. Because the NHI program is single-player and compulsory for citizens and legal residents, it covers almost all 23 million residents in Taiwan. By the end of 2014, > 99.6% of the population of Taiwan was enrolled in the NHI, and the contract rate was >93% [17]. In the NHIRD, disease is coded according to the International Classification of Diseases, Revision 9, Clinical Modification (ICD-9-CM). Patient information in the NHIRD is de-identified. The study protocol was approved by the Chang-Gung Memorial Hospital Research Ethics Committee (Institutional Review Board number: 103-6439B).

Patients with specific diagnosis codes (one inpatient or two outpatient codes) for HCV (070.41, 070.44, 070.51, 070.54, 070.70, 070.71, V02.62) recorded between 1 January 2003 and 31 December 2013 were identified.

### Eligibility criteria and identification of study cohorts

First, we excluded HCV-infected patients without a claims-based diagnosis of CKD (Additional file 1) including dialysis (ICD-9-CM code 585) and registered in the Catastrophic Illness Patient Database (CIPD). Second, we excluded patients <20 years old and >80 years, co-infected with hepatitis B virus (HBV) or comorbidities possibly associated with interferon intolerance: stroke, myocardial infarction, heart failure, hepatic decompensation (esophageal or gastric varices, hepatic encephalopathy, hepatorenal syndrome, portal hypertension, and ascites), hepatic surgery or liver transplantation, all malignancies, catastrophic illness for autoimmune disease, and severe psychosis/mental disorder (Additional file 1). Additionally, to reduce survival bias, all patients who had died, had clinical outcomes, or had been lost to follow-up within 6 months after the index date were excluded. We finally enrolled 9872 HCV-infected patients with CKD in the study.

**Fig. 1:**
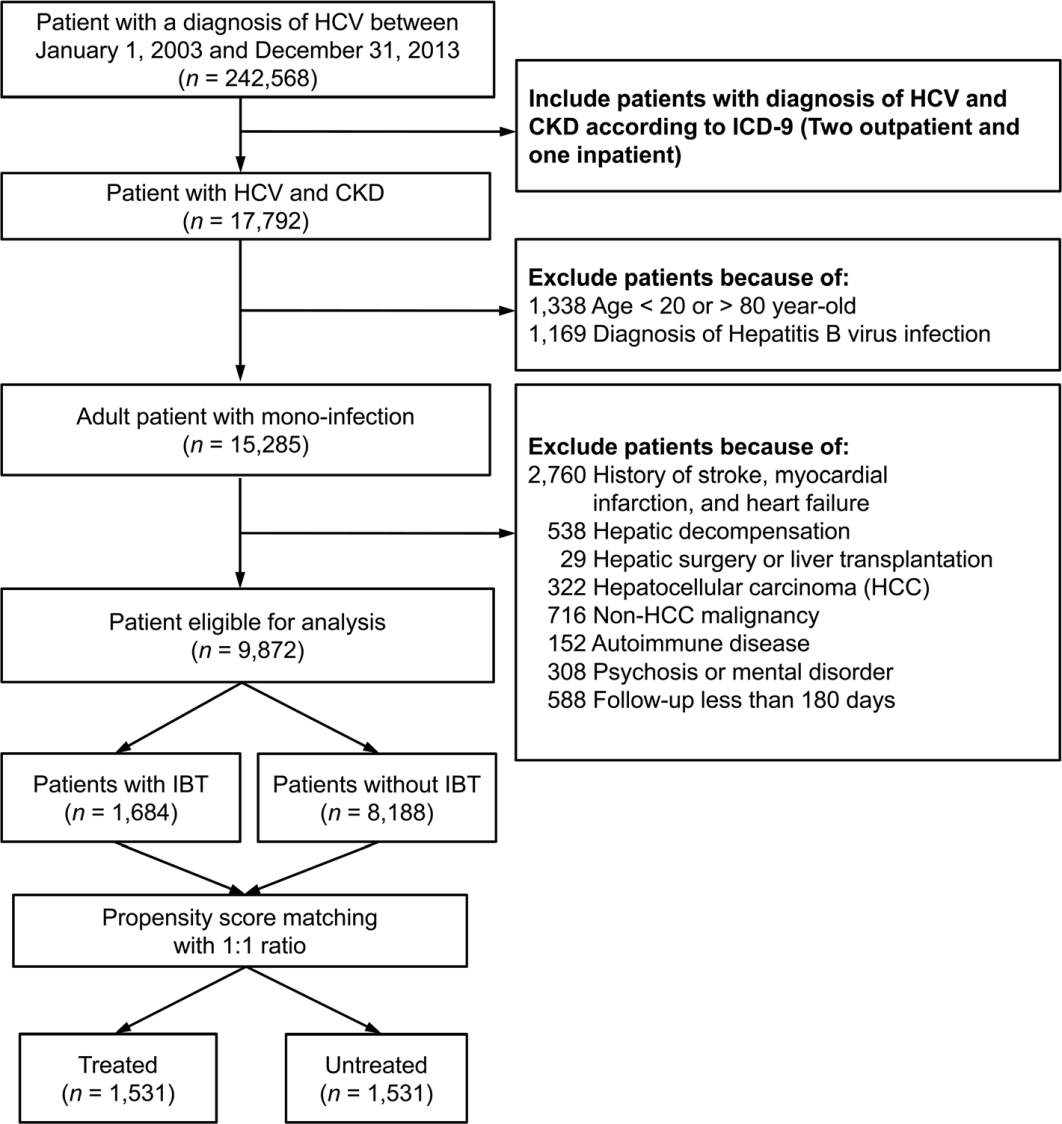
Flowchart of using stepwise exclusion to identify and enroll patients

Patients undergoing anti-viral pegylated interferon (Peg-IFN) treatment (*n* = 1684) were assigned to the IBT-treated cohort and patients, who had never undergone interferon or ribavirin treatment throughout the study period (*n* = 8188) were assigned to the IBT-untreated cohort. Each patient in the treated cohort was matched with one patient in the untreated cohort using the probability calculated by logistic regression based on the following confounding variables: gender, age, hospital levels, comorbidities, dialysis status, and medications (listed in Table 1). Figure 1 shows a detailed flowchart of the study. The index date was set as the date when HCV was first diagnosed in the untreated cohort and as the date when interferon was first prescribed in the treated cohort.

**Table 1:** Characteristics of the study patients before and after propensity score matching

	Before matching			After matching		
Variable	Treated cohort (*n* = 1684)	Untreated cohort (*n* = 8188)	*P*-value	Treated cohort (*n* = 1531)	Untreated cohort (*n* = 1531)	*P*-value
Characteristic and comorbidity						
Gender			<0.001			0.476
Male	1090 (64.7)	4359 (53.2)		978 (63.9)	959 (62.6)	
Female	594 (35.3)	3829 (46.8)		553 (36.1)	572 (37.4)	
Age in years	57.7 ± 10.2	60.5 ± 11.8	<0.001	58.1 ± 10.2	58.4 ± 11.6	0.406
Age group			<0.001			0.603
20 ~ 40 years	97 (5.8)	468 (5.7)		88 (5.7)	88 (5.7)	
40 ~ 60 years	852 (50.6)	3225 (39.4)		747 (48.8)	720 (47.0)	
60 ~ 80 years	735 (43.6)	4495 (54.9)		696 (45.5)	723 (47.2)	
Income, NTD per month			<0.001			0.849
< 10,000	290 (17.2)	1980 (24.2)		282 (18.4)	302 (19.7)	
10,000 ~ 19,999	112 (6.7)	600 (7.3)		105 (6.9)	100 (6.5)	
20,000 ~ 29,999	768 (45.6)	3679 (44.9)		704 (46.0)	680 (44.4)	
30,000 ~ 39,999	199 (11.8)	678 (8.3)		168 (11.0)	168 (11.0)	
≥ 40,000	315 (18.7)	1251 (15.3)		272 (17.8)	281 (18.4)	
Urbanization level			0.249			0.713
1, most urbanized	382 (22.7)	1711 (20.9)		338 (22.1)	344 (22.5)	
2	481 (28.6)	2280 (27.8)		441 (28.8)	430 (28.1)	
3	537 (31.9)	2758 (33.7)		495 (32.3)	518 (33.8)	
4, least urbanized	284 (16.9)	1439 (17.6)		257 (16.8)	239 (15.6)	
Hospital levels			<0.001			0.942
Medical center	590 (35.0)	1779 (21.7)		528 (34.5)	523 (34.2)	
Region hospital	814 (48.3)	2706 (33.0)		725 (47.4)	731 (47.7)	
District hospital	174 (10.3)	1892 (23.1)		172 (11.2)	178 (11.6)	
Clinics	106 (6.3)	1811 (22.1)		106 (6.9)	99 (6.5)	
Medical history						
Diabetes mellitus	717 (42.6)	2913 (35.6)	<0.001	644 (42.1)	651 (42.5)	0.798
Hypertension	1004 (59.6)	4479 (54.7)	<0.001	915 (59.8)	914 (59.7)	0.971
Dyslipidemia	203 (12.1)	1179 (14.4)	0.012	191 (12.5)	191 (12.5)	1.000
Liver cirrhosis	187 (11.1)	566 (6.9)	<0.001	161 (10.5)	179 (11.7)	0.301
COPD	78 (4.6)	544 (6.6)	0.002	74 (4.8)	87 (5.7)	0.293
PAD	42 (2.5)	262 (3.2)	0.127	39 (2.5)	46 (3.0)	0.441
Thyroid disease	42 (2.5)	132 (1.6)	0.012	32 (2.1)	37 (2.4)	0.543
On dialysis	253 (15.0)	3102 (37.9)	<0.001	251 (16.4)	228 (14.9)	0.469
Medication						
Anti-platelet agents	251 (14.9)	1458 (17.8)	0.004	235 (15.3)	237 (15.5)	0.920
Oral hypoglycemia agents	592 (35.2)	2136 (26.1)	<0.001	529 (34.6)	535 (34.9)	0.820
Insulin	202 (12.0)	986 (12.0)	0.957	189 (12.3)	193 (12.6)	0.827
Statin	113 (6.7)	909 (11.1)	<0.001	111 (7.3)	116 (7.6)	0.730
NSAID	661 (39.3)	3502 (42.8)	0.008	609 (39.8)	597 (39.0)	0.657
COX-II inhibitors	90 (5.3)	767 (9.4)	<0.001	88 (5.7)	88 (5.7)	1.000
Beta blockers	416 (24.7)	2060 (25.2)	0.694	386 (25.2)	397 (25.9)	0.649
Diuretics	93 (5.5)	934 (11.4)	<0.001	93 (6.1)	100 (6.5)	0.603
Spironolactone	32 (1.9)	217 (2.7)	0.074	32 (2.1)	41 (2.7)	0.286
Steroid	156 (9.3)	897 (11.0)	0.041	149 (9.7)	151 (9.9)	0.903
Anti-HTN agent						
ACEi/ARB	652 (38.7)	2755 (33.6)	<0.001	600 (39.2)	593 (38.7)	0.795
CCB (Dihydropyridine CCB)	486 (28.9)	2659 (32.5)	0.004	447 (29.2)	436 (28.5)	0.661
Others (include alpha blocker)	106 (6.3)	645 (7.9)	0.026	101 (6.6)	103 (6.7)	0.885
Number of anti-HTN agents			0.260			0.979
0	808 (48.0)	4032 (49.2)		725 (47.4)	731 (47.7)	
1	546 (32.4)	2460 (30.0)		499 (32.6)	500 (32.7)	
2	292 (17.3)	1489 (18.2)		272 (17.8)	268 (17.5)	
≥ 3	38 (2.3)	207 (2.5)		35 (2.3)	32 (2.1)	
Follow-up (years)	3.6 ± 2.6	5.0 ± 3.0	<0.001	3.8 ± 2.7	3.6 ± 2.6	0.070

*ACEi* angiotensin converting enzyme inhibitor, *ARB* angiotensin receptor blocker, *CCB* calcium channel blockers, *COX-II* Cyclooxygenase II, *COPD* chronic obstructive pulmonary disease, *HTN* hypertension, *NSAID* non-steroidal anti-inflammatory drug, *NTD* New Taiwan Dollar, *PAD* peripheral arterial disease

### Definition of Comorbidities and drug exposure

The comorbidities were diagnosed and then recorded in NHIRD using ICD-9-CM codes. All of the comorbidities listed in this study were confirmed on at least two consecutive clinic visits or whenever the patient had been hospitalized before the index date. In addition, some comorbidities were also reconfirmed using long-term associated medications. The definitions of the comorbidities are listed in the Additional file 1. Personal socio-economic status was divided to five levels according to monthly income (<10,000, 10,000 ~ 19,999, 20,000 ~ 29,999, 30,000 ~ 39,999, ≥ 40,000 New Taiwan Dollar [NTD]). Based on Tzeng’s classification [18], we modified the score and categorized all Taiwan areas into four levels (most to least urbanization). Drug information—drug names, date of prescription, and duration of use—was obtained from the outpatient pharmacy prescription database. Long-term drug use was defined as a prescription for at least 6 months after the index date.

The therapeutic duration of IBT generally ranged between 16 and 48 weeks [19] based on viral information (genotype and virological response) and the patient’s ability to tolerate the treatment. According to the response-guided trial [20] and Taiwan Health insurance regulations, we will check early viral response (EVR) at week 12 and premature termination (before 16 weeks) usually results from either intolerance to side effects or failure of early virological response or death. Treated patients were consequently subgrouped based on whether or not their antiviral treatment was ≥16 weeks or <16 weeks. “Incompletely treated” cohort means “treated <16 weeks”.

### Definition and ascertainment of outcomes

Enrollees were observed until occurrence of the clinical outcomes, death, or 31 December 2013, whichever came first. The clinical outcomes were all-cause mortality, acute myocardial infarction, ischemic stroke, and hemorrhagic stroke, all of which were confirmed based on the primary diagnosis of the index hospitalization. These outcomes have been validated before and confirmed as highly accuracy [8, 9]. The other outcome was new-onset dialysis, which was defined as first-time and necessary maintenance dialysis by certification in the CIPD registry.

### Statistical analysis

We performed propensity score matching (PSM) before comparing the treated cohort with the untreated cohort because there was substantial difference between the two cohorts that might confound the result [21]. Covariates to be calculated in the propensity score included index year (as the year of IBT) and variables listed in Table 1 (except for the follow up year). Propensity score indicates the predicted probability of the logistic regression and each patient in the treated cohort was matched with a corresponding patient in the untreated cohort. To warranty the quality of matching, the tolerance level on the maximum propensity score distance (caliper) was set as 0.2 × standard deviation of the propensity score under the greedy nearest neighbor matching algorithm.

We compared the patient’s characteristics between groups (i.e. treated cohort vs. untreated cohort) using independent sample t-test for continuous variable or using chi-square test for categorical variable. The incidence of outcome (mortality, acute myocardial infarction, ischemic stroke, hemorrhagic stroke, and new onset dialysis) was estimated using incidence density as number of event per 1000 person-years since entry of index date. The time-to-event between the two cohorts was compared using a Cox proportional hazard analysis adjusted for the propensity score. In an additional subgroup analysis, we stratified the treated cohort into two groups (incompletely treated <16 weeks and treated ≥16 weeks) and compared the time-to-event between the groups using a log-rank test. Data analysis was performed with SAS Version 9.3 (SAS Institute, Cary, NC).

## Results

### Baseline characteristics of the study population

We identified 17,792 HCV-infected patients with CKD among the 242,568 patients diagnosed with HCV between January 1, 2003, and December 31, 2013. After patients who did not meet our inclusion criteria had been excluded, 1684 patients (mean age: 57.7 ± 10.2 years; men: 65%) were in the pre-PSM treated group, and 8188 (mean age: 60.5 ± 11.8 years; men: 53%) were in the pre-PSM untreated group (Table 1). Most patients also accepted antiviral therapy at medical center and regional hospital. Despite higher income status in pre-PSM treated group, the residential urbanization level was insignificantly different among both cohorts. The pre-PSM treated HCV patients had a higher prevalence of diabetes and hypertension and they tended to receive oral hypoglycemia agents and ACEi/ARB other than CCB. Only 15% of the patients in the pre-PSM treated cohort were on dialysis, but 38% in the pre-PSM untreated cohort were. The prevalence of liver cirrhosis without hepatic decompensation was higher in the pre-PSM treated than in untreated cohorts (11.1% vs. 6.9%, *P* < 0.001). Finally, after 1:1 PSM, the distribution of demographic factors, hospital levels, comorbidities, and associated medications were not significantly different between the two cohorts (Table 1).

### Outcomes during the 10-year longterm follow up

**Fig. 2:**
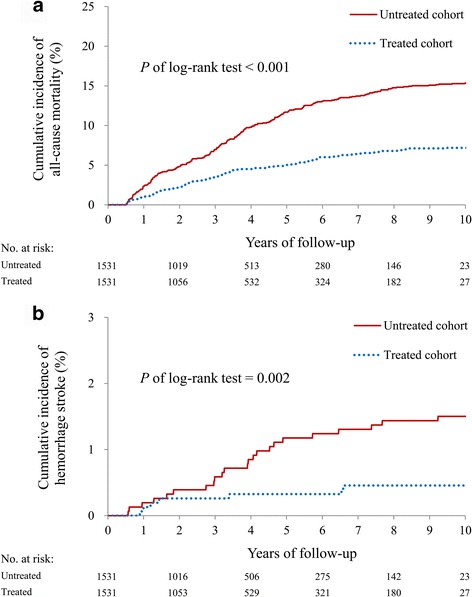
**a** All-cause mortality survival curves in the treated and untreated cohorts; **b** incidence density of hemorrhagic stroke between the treated and untreated cohorts

Outcomes were assessed after 1:1 PSM, which yielded 1531 treated patients and 1531 untreated patients. The annual incidence of all-cause mortality was apparently higher in the untreated cohort (incidence density [ID], 42.89; 95% confidence interval [CI], 37.43–48.35) than in the treated cohort (ID: 19.00; 95% CI: 15.45–22.55), which was significantly different (adjusted hazard ratio [aHR], 0.45; 95% CI: 0.36–0.56, *P* < 0.001). The risk of secondary outcomes, including acute myocardial infarction and ischemic stroke, were comparable between the two cohorts. Noticeably, the ID of hemorrhagic stroke was 1.21 (95% CI: 0.31–2.11) in the treated cohort and 4.19 (95% CI: 2.48–5.91) in the untreated cohort, a significantly different risk reduction in the treated cohort (aHR: 0.31; 95% CI, 0.13–0.71, *P* = 0.006). The cumulative incidence between the two cohorts for all-cause mortality is plotted in Fig. 2a, and for hemorrhagic stroke in Fig. 2b.

An evaluation of the effect of dialysis on IBT showed that all-cause mortality reduction was less efficacy for HCV patients on dialysis than for HCV patients not on dialysis (aHR: 0.80 vs. 0.39; *P* for interaction = 0.018). The effect of IBT on the other three outcomes was comparable between the two cohorts (Additional file 2).

### Effect of incomplete IBT on clinical outcomes

**Fig. 3:**
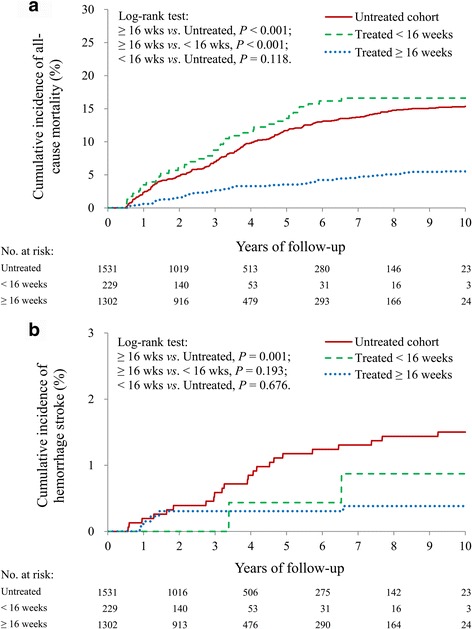
**a** All-cause mortality survival curves in the “treated ≥16 weeks” and incompletely treated (<16 weeks) and untreated cohorts; **b** incidence density of hemorrhagic stroke in the three cohorts based on the duration of therapy

We stratified the treated cohort into two groups (incompletely treated <16 weeks and treated 16 ≥ weeks). The mortality rate was higher in the incompletely treated (<16 weeks) cohort than in the treated (≥16 weeks) cohort (*P* < 0.001), but it was comparable with the untreated cohort (*P* = 0.118) (Fig. 3a). The risk of hemorrhagic stroke in the incompletely treated cohort was comparable with the risks in the other two groups (Fig. 3b).

Patients in the incompletely treated cohort tended to be older, have a higher prevalence of liver cirrhosis and dialysis, took more beta blockers, number of anti-HTN agents and diuretics, including spironolactone, a potassium-sparing diuretic (Additional file 3).

### Renoprotective effect in non-dialytic patients

**Fig. 4:**
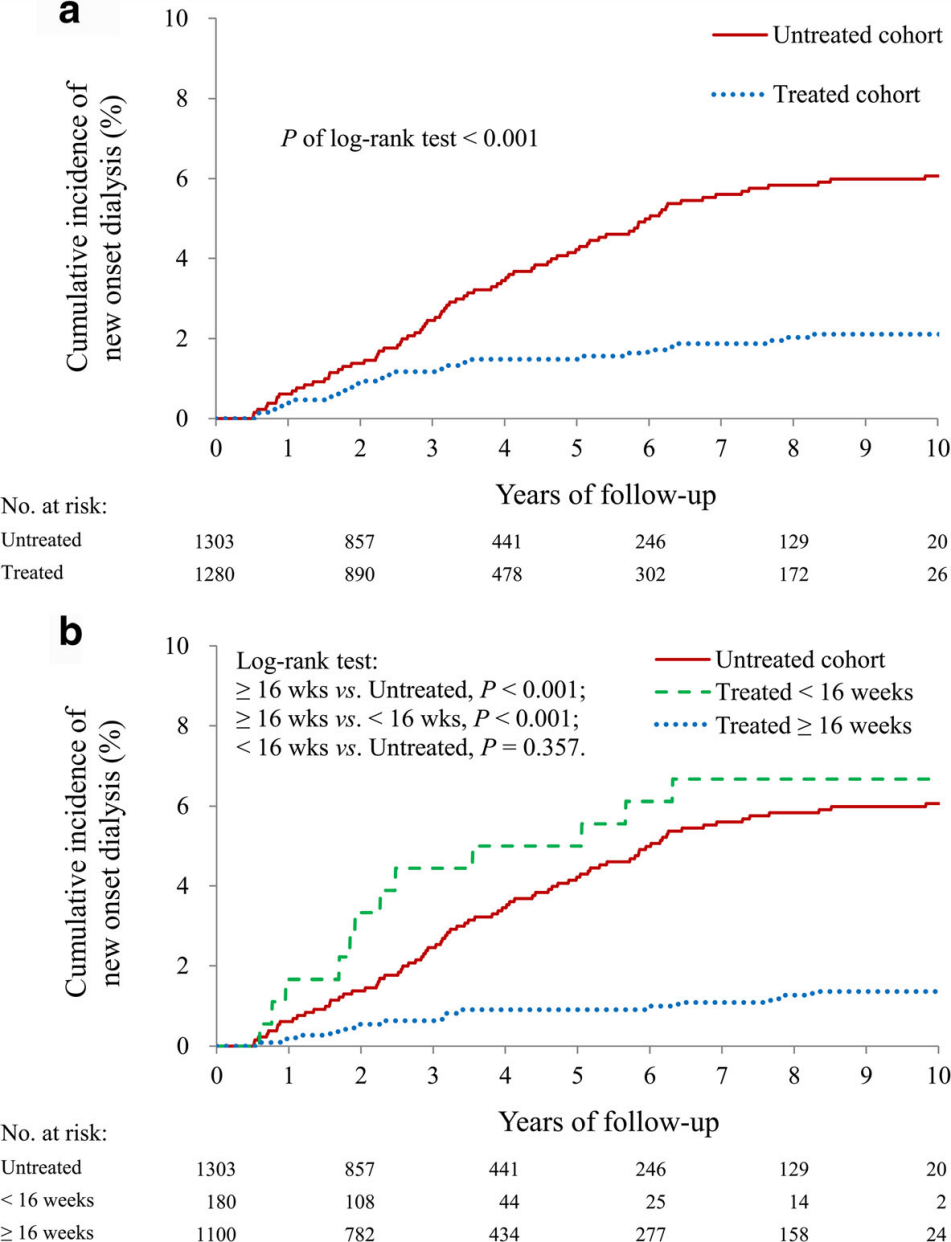
**a** Incidence density of new-onset dialysis between the treated and untreated cohorts in CKD patients without dialysis; **b** the treated cohort was subgrouped by therapeutic duration

After patients on dialysis before the index date had been excluded, antiviral therapy was associated with a lower risk of new-onset dialysis (aHR: 0.31; 95% CI: 0.20–0.48, *P* < 0.001) (Table 2 and Fig. 4a). Additionally, the incidence of new-onset dialysis in the incompletely treated (<16 weeks) cohort was greater than that in the treated (≥16 weeks) cohort (*P* < 0.001), but it was comparable with that of the untreated cohort (*P* = 0.357) (Fig. 4b).

**Table 2:** Event numbers and incidence density (ID) of the outcomes between the study cohorts

Variable	Treated cohort (*n* = 1531)	Untreated cohort (*n* = 1531)	*P*-value‡
All-cause mortality			
Follow-up (years), mean ± SD	3.78 ± 2.66	3.61 ± 2.57	
Event number, n (%)	110 (7.18)	237 (15.48)	
ID (95% CI)^a^	19.00 (15.45–22.55)	42.89 (37.43–48.35)	
Hazard ratio (95% CI)	0.45 (0.36–0.56)	Reference	<0.001
Acute myocardial infarction			
Follow-up (years), mean ± SD	3.77 ± 2.66	3.59 ± 2.56	
Event number, n (%)	12 (0.78)	13 (0.85)	
ID (95% CI)^a^	2.08 (0.90–3.26)	2.36 (1.08–3.65)	
Hazard ratio (95% CI)	0.92 (0.42–2.01)	Reference	0.826
Ischemic stroke			
Follow-up (years), mean ± SD	3.75 ± 2.64	3.58 ± 2.56	
Event number, n (%)	17 (1.11)	18 (1.18)	
ID (95% CI)^a^	2.96 (1.55–4.37)	3.28 (1.77–4.80)	
Hazard ratio (95% CI)	0.91 (0.47–1.76)	Reference	0.774
Hemorrhagic stroke			
Follow-up (years), mean ± SD	3.77 ± 2.66	3.58 ± 2.55	
Event number, n (%)	7 (0.46)	23 (1.50)	
ID (95% CI)^a^	1.21 (0.31–2.11)	4.19 (2.48–5.91)	
Hazard ratio (95% CI)	0.31 (0.13–0.71)	Reference	0.006
New onset dialysis (*n* = 2583)^b^			
Study number	1280	1303	
Follow-up (years), mean ± SD	3.93 ± 2.75	3.61 ± 2.61	
Event number, n (%)	27 (2.11)	79 (6.06)	
ID (95% CI)^a^	5.37 (3.34–7.39)	16.78 (13.08–20.48)	
Hazard ratio (95% CI)	0.31 (0.20–0.48)	Reference	<0.001

*SD* standard deviation, *CI* confidence interval

^a^Incidence density (ID): event numbers per 1000 person-years

^b^Patients who were under dialysis were excluded

‡Adjusted for propensity score

## Discussion

This is the first nationwide cohort study to investigate long-term outcomes of patients with HCV and comorbid CKD treated with IBT, which demonstrated significant benefits on all-cause mortality and new-onset dialysis compatible with previous non-CKD HCV analysis [8, 9]. We also found a markedly lower incidence for hemorrhagic stroke among treated patients regardless of dialysis, but not in untreated patients. Although many patients cannot tolerate IBT and stop the therapy, the survival benefit for treated HCV-infected patients with comorbid CKD is higher than that for untreated patients.

### Survival benefits of IBT in HCV-infected patients with comorbid CKD

High mortality of chronic HCV infection is attributable not only to hepatic decompensation, liver cirrhosis, and hepatocellular carcinoma (HCC), but also to multiple extrahepatic complications, which can be ameliorated by using complete IBT in HCV-infected patients without CKD [8, 9]. Because of the genotype distribution of 50% for HCV-1 and 45% for HCV-2 in Taiwan [19], sustained viral response (SVR) for HCV with comorbid CKD or dialysis was 50–64% [10, 11]. Hsu et al. [8] demonstrated a small reduction of cardiovascular events (<20%) in a treated cohort, which was comparable with the findings of a study on diabetes [9]. However, our study showed neural cardiovascular effects of antiviral therapy in HCV-infected patients with CKD, which might be related to drug intolerability, IBT inefficacy, and severe atherosclerosis in CKD patients, but not in patients without CKD.

IBT might reduce all-cause mortality because it ameliorates the hepatic complications of decompensation, liver cirrhosis, and HCC, or non-hepatic problems like a tendency to hemorrhage, immunocompromised sepsis, advanced renal impairment, hemodialysis, etc.

### Cerebrovascular protection of IBT in patients with chronic HCV and CKD

Our study showed a significant reduction of hemorrhagic stroke (10-year hemorrhagic stroke incidence rate: untreated = 1.5% vs. treated = 0.46%), which is comparable to the findings of Arase et al. [22] on HCV clearance (10-year incidence rate in HCV non-clearance = 1.1% vs. clearance = 0.4%; *P* = 0.02). However, their study lacked renal function data before IBT and background matching between cohorts. Despite conventional factors like age, being male, smoking, hypertension, diabetes, and drinking alcohol [23], HCV contributed to hemorrhagic stroke [24, 25], liver cirrhosis [26–28], and unstable hemostasis [29]. Moreover, HCV was critically involved in systemic atherosclerosis, vascular remodeling, ectasia change, and microaneurysms, which manifested as cerebral microbleeds (CMBs) in the MRIs of patients with advanced hepatic fibrosis [30]. In addition to viral replication and HCV receptor expression in microvascular endothelium [31], neurological vasculitis was highly prevalent with mixed cryoglobulinemia in 25–60% of HCV-infected patients [32, 33]. Both consequences increased the risk of microvascular destruction and disturbance of the brain–blood-barrier related to spontaneous hemorrhagic events. Indeed, the protection of complete IBT against hemorrhagic stroke comes not only because it ameliorates advanced liver cirrhosis, but also because it attenuates extrahepatic immunological complications.

Both the brain and the kidneys are the end-organs for high blood pressure and autoregulation in hypertensive patients [34]. Thus, CKD might be another risk factor of hemorrhagic stroke because of an altered response to antiplatelet agents, platelet dysfunction, endothelial hyper-permeability [35], microvascular dysregulation [36], and occult CMBs [37], Our subgroup analysis showed that IBT promised a greater reduction of new-onset dialysis and constant protection against hemorrhagic stroke, regardless of whether the patient was on dialysis, and that antiviral therapy provided many benefits for the liver, kidney, and brain triangle.

### Effect of incomplete IBT on clinical outcomes

According to a meta-analysis [11], the dropout rate in patients on dialysis was 18%. Our finding was 14.4% for incompletely treated patients. Both rates were higher than that of Hsu et al. [8, 9]: 10.4% in a general population without CKD. Hsu et al. showed that patients who underwent incomplete IBT were older, as were our patients, who also had more liver cirrhosis and more dialysis. Additionally, many incompletely treated patients had been prescribed diuretics because they had more fluid overloading or ascites due to hepatic decompensation. Despite the loss of benefits because of incomplete IBT, our analysis and other studies also found slightly higher mortality in patients treated for <16 weeks, possibly because they had more critical comorbidities, marked anemia, immunocompromised status, or malnutrition. In addition, interferon might cause adverse effects or complications, including heart failure and infection [38]. Because of high mortality in the incompletely treated group, identifying the precipitating factors before starting IBT is critical.

### Limitations

This study has some limitations. First, details of the HCV genotype, IL-28B polymorphism, virus load, viral response, serum creatinine or eGFR, duration of renal impairment, duration of dialysis, platelet and coagulation function, echogenic study, degree of hepatic fibrosis, body weight, and personal lifestyles are not included in the Taiwan NHIRD. However, by carefully excluding confounding comorbidities and estimating associated variables, including medications, we obtained information that contributed to outcomes. Second, the misdiagnoses of CKD or cirrhosis might occur because of the ICD-9 codes used in several NHIRD-based nationwide cohort studies, but we used the standard methodology (1 inpatient or 2 outpatient diagnosis codes) to identify CKD patients in the NHIRD. Underdiagnosis of cirrhosis might also occur in CKD or dialysis population due to relatively low aminotransferase [39]. Moreover, our study tried to the gap of longterm outcomes in HCV-infected patients with CKD, which is difficult to do in clinical researched because of individual decision making unpredictable dose adjustments at various stages. Third, alcohol consumption might be involved in hemorrhagic stroke because of alcohol-induced hypertension and coagulation disorders, whether or not the patient has cirrhosis [23]. Moreover, we had carefully excluded alcohol-related liver cirrhosis the number of confounding factors as much as possible. Fourth, variable doses of ribavirin according to renal function, body weight or drug tolerance might influence effect of viral eradication, but all treated patients would receive guided interferon therapy. Fifth, the effects of events that occurred within the therapeutic course (<6 months) and their association with clinical benefits, adverse side effects of drugs, and underlying comorbidities were not clear. Therefore, we excluded patients who had died, or had a myocardial infarction, ischemic stroke, hemorrhagic stroke, new-onset dialysis, or who were lost to follow-up within 6 months, which is different from other studies [8, 9].

## Conclusion

Despite neural effect on AMI and IS, our study showed that antiviral therapy might provide benefits on all-cause mortality, late dialysis, and hemorrhagic stroke in those HCV infected patients with CKD. In addition, DAA, as an agent with higher SVR and better tolerance, would enhance effective viral eradication and comprehensive cardiovascular protection for the difficultly treated population.

## Additional files

Additional file 1: Appendix of ICD-9-CM codes. List of all ICD-9-CM codes used for diagnosis in the current study. (DOC 42 kb)

Additional file 2: Subgroup analyses of IBT efficacy. The effect of IBT on all outcomes was comparable between the two cohorts regarding dialysis. (TIFF 1004 kb)

Additional file 3: Characteristics comparison by different IBT courses. Characteristics differences of HCV-infected patients receiving IBT ≥ 16 or <16 weeks after propensity score matching. (DOC 79 kb)

## Abbreviations

ACEi: Angiotensin converting enzyme inhibitor
aHR: Adjusted hazard ratio
ARB: Angiotensin receptor blocker
CI: Confidence intervals
CKD: Chronic kidney disease
COPD: Chronic obstructive pulmonary disease
COX-II: Cyclooxygenase-2
DM: Diabetes mellitus
ESRD: End-stage renal disease
HCV: Hepatitis C virus
HR: Hazard ratio
HTN: Hypertension
IBT: Interferon-based therapy
ICD-9-CM: International Classification of Diseases, Ninth Revision, Clinical Modification
NHI: National Health Insurance
NHIRD: National health Insurance Research Database
NSAID: Non-steroidal anti-inflammatory drug
PAD: Peripheral arterial disease
SD: Standard deviation
