# Supplementary material for: Add-on neurological benefits of antiviral therapy in HCV patients with chronic kidney disease — a nationwide cohort study
Source: BMC Gastroenterol. 2017 Aug 16;17:99. doi: 10.1186/s12876-017-0653-2 (PMC5559858; doi:10.1186/s12876-017-0653-2)
Supplement: Supplementary file 1 — Appendix of ICD-9-CM codes. List of all ICD-9-CM codes used for diagnosis in the current study. (DOC 42 kb) [file 12876_2017_653_MOESM1_ESM.doc]

Appendix. ICD-9-CM code used for diagnosis in the current study

| Variable | Code |
| --- | --- |
| Hepatitis C virus infection | 070.41, 070.44, 070.51, 070.54, 070.70, 070.71, V02.62 |
| Hepatitis B virus infection | 070.20, 070.22, 070.30, 070.32, V02.61 |
| Chronic kidney disease | 580.xx–589.xx, 403.xx–404.xx,  016.0x, 095.4x, 236.9x, 250.4x, 274.1x, 442.1x, 447.3x, 440.1x, 572.4x, 642.1x, 646.2x, 753.1x,  283.11, 403.01, 404.02, 446.21 |
| Old stroke | 430.xx–437.xx |
| Old myocardial infarction | 410.xx, 412.xx |
| Heart failure | 428.xx |
| Hepatic decompensation | 456.0x–456.2x, 572.2x, 572.3x, 572.4x, 789.5x |
| Hepatic transplantation | V42.7 |
| Hepatocellular carcinoma | 155.xx |
| Malignancy | 140.xx–208.xx (Catastrophic illness card) |
| Autoimmune disease | 710.0, 710.1, 710.2, 714.0,  714.30–714.33, 710.3, 710.4,  446.0, 446.2, 446.4, 446.5, 446.7, 443.1 |
| Psychosis or mental disorder | 295.xx–297.xx |
| Diabetes mellitus | 250.xx (combined drug) |
| Hypertension | 401.xx–405.xx (combined drug) |
| Dyslipidemia | 272.xx (combined drug) |
| Liver cirrhosis | 571.2, 571.5, 571.6 |
| Chronic obstructive pulmonary disease | 491.xx, 492.xx, 496.xx |
| Peripheral arterial disease | 440.0x, 440.2x, 440.3x, 440.8x, 440.9x, 443.xx, 444.0x, 444.22, 444.8x, 447.8x, 447.9x |
| Thyroid disease | 242.xx–245.xx |
| Acute myocardial infarction | 410.xx |
| Ischemic stroke | 433.xx–437.xx |
| Hemorrhagic stroke | 430.xx–432.xx |
| Dialysis | 585.xx (Catastrophic illness card) |
