# Supplementary figures and images for: Add-on neurological benefits of antiviral therapy in HCV patients with chronic kidney disease — a nationwide cohort study
Source: BMC Gastroenterol. 2017 Aug 16;17:99. doi: 10.1186/s12876-017-0653-2 (PMC5559858; doi:10.1186/s12876-017-0653-2)

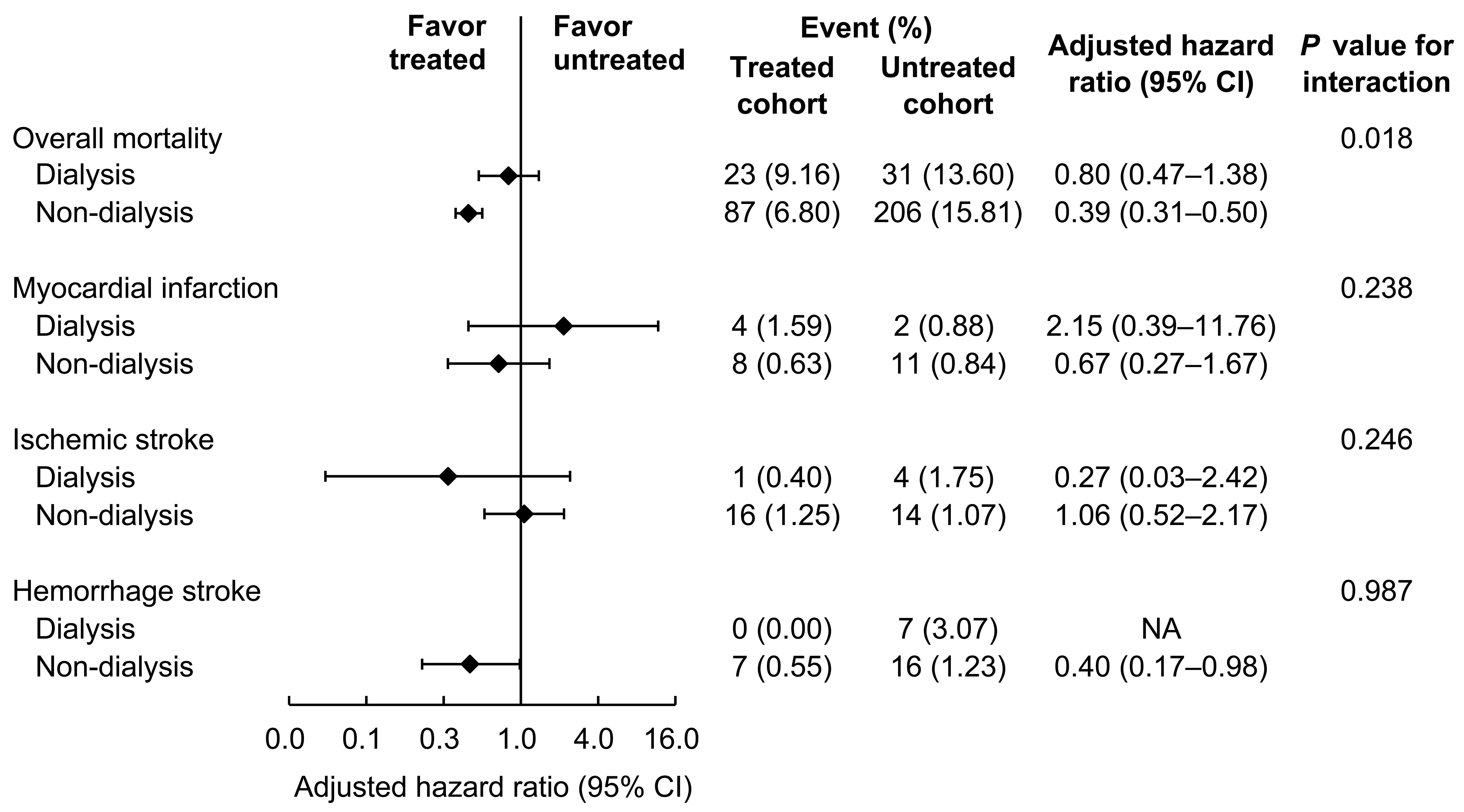

Supplement: Supplementary file 2 — Subgroup analyses of IBT efficacy. The effect of IBT on all outcomes was comparable between the two cohorts regarding dialysis. (TIFF 1004 kb) [file 12876_2017_653_MOESM2_ESM.tif]
