# Supplementary material for: Add-on neurological benefits of antiviral therapy in HCV patients with chronic kidney disease — a nationwide cohort study
Source: BMC Gastroenterol. 2017 Aug 16;17:99. doi: 10.1186/s12876-017-0653-2 (PMC5559858; doi:10.1186/s12876-017-0653-2)
Supplement: Supplementary file 3 — Characteristics comparison by different IBT courses. Characteristics differences of HCV-infected patients receiving IBT ≥ 16 or <16 weeks after propensity score matching. (DOC 79 kb) [file 12876_2017_653_MOESM3_ESM.doc]

Additional file 3. Characteristics of HCV-infected patients receiving IBT ≥ 16 or < 16 weeks after propensity score matching

| Variable | Treated ≥ 16 weeks  (*n* = 1,302) | Treated < 16 weeks  (*n* = 229) | *P*-value |
| --- | --- | --- | --- |
| Characteristic and comorbidity |  |  |  |
| Gender |  |  | 0.579 |
| Male | 828 (63.6) | 150 (65.5) |  |
| Female | 474 (36.4) | 79 (34.5) |  |
| Age in years | 57.5±10.1 | 61.3±10.3 | <0.001 |
| Age group |  |  | <0.001 |
| 20~40 years | 80 (6.1) | 8 (3.5) |  |
| 40~60 years | 664 (51.0) | 83 (36.2) |  |
| 60~80 years | 558 (42.9) | 138 (60.3) |  |
| Income, NTD per month |  |  | 0.048 |
| < 10,000 | 227 (17.4) | 55 (24.0) |  |
| 10,000~19,999 | 94 (7.2) | 11 (4.8) |  |
| 20,000~29,999 | 597 (45.9) | 107 (46.7) |  |
| 30,000~39,999 | 142 (10.9) | 26 (11.4) |  |
| ≥ 40,000 | 242 (18.6) | 30 (13.1) |  |
| Urbanization level |  |  | 0.495 |
| 1, most urbanized | 282 (21.7) | 56 (24.5) |  |
| 2 | 383 (29.4) | 58 (25.3) |  |
| 3 | 416 (32.0) | 79 (34.5) |  |
| 4, least urbanized | 221 (17.0) | 36 (15.7) |  |
| Hospital levels |  |  | 0.251 |
| Medical center | 460 (35.3) | 68 (29.7) |  |
| Region hospital | 611 (46.9) | 114 (49.8) |  |
| District hospital | 146 (11.2) | 26 (11.4) |  |
| Clinics | 85 (6.5) | 21 (9.2) |  |
| Medical history |  |  |  |
| Diabetes mellitus | 545 (41.9) | 99 (43.2) | 0.698 |
| Hypertension | 772 (59.3) | 143 (62.4) | 0.370 |
| Dyslipidemia | 159 (12.2) | 32 (14.0) | 0.457 |
| Liver cirrhosis | 124 (9.5) | 37 (16.2) | 0.003 |
| COPD | 63 (4.8) | 11 (4.8) | 0.982 |
| PAD | 32 (2.5) | 7 (3.1) | 0.596 |
| Thyroid disease | 28 (2.2) | 4 (1.7) | 0.694 |
| On dialysis | 202 (15.5) | 49 (21.4) | 0.003 |
| Medication |  |  |  |
| Anti-platelet agents | 195 (15.0) | 40 (17.5) | 0.335 |
| Oral hypoglycemia agents | 447 (34.3) | 82 (35.8) | 0.665 |
| Insulin | 152 (11.7) | 37 (16.2) | 0.057 |
| Statin | 90 (6.9) | 21 (9.2) | 0.224 |
| NSAID | 517 (39.7) | 92 (40.2) | 0.894 |
| COX-II inhibitors | 71 (5.5) | 17 (7.4) | 0.237 |
| Beta blockers | 312 (24.0) | 74 (32.3) | 0.007 |
| Diuretics | 58 (4.5) | 35 (15.3) | <0.001 |
| Spironolactone | 15 (1.2) | 17 (7.4) | <0.001 |
| Steroid | 128 (9.8) | 21 (9.2) | 0.756 |
| Anti-HTN agent |  |  |  |
| ACEi/ARB | 501 (38.5) | 99 (43.2) | 0.174 |
| CCB (Dihydropyridine CCB) | 367 (28.2) | 80 (34.9) | 0.038 |
| Others (include alpha blocker) | 81 (6.2) | 20 (8.7) | 0.158 |
| Number of anti-HTN agents |  |  | 0.085 |
| 0 | 628 (48.2) | 97 (42.4) |  |
| 1 | 425 (32.6) | 74 (32.3) |  |
| 2 | 223 (17.1) | 49 (21.4) |  |
| ≥ 3 | 26 (2.0) | 9 (3.9) |  |
| Follow-up (years) | 3.9±2.7 | 3.1±2.3 | <0.001 |

ACEi, angiotensin converting enzyme inhibitor; ARB, angiotensin receptor blocker; CCB, calcium channel blockers; COX-II, Cyclooxygenase II; COPD, chronic obstructive pulmonary disease; HTN, hypertension; NSAID, non-steroidal anti-inflammatory drug; NTD, New Taiwan Dollar; PAD, peripheral arterial disease.
